# Breaking the maternity mold: navigating the return to work and challenging rigid maternal beliefs through an online psychological intervention

**DOI:** 10.3389/fgwh.2024.1266162

**Published:** 2024-04-04

**Authors:** Sebastiano Rapisarda, Alessandro De Carlo, Elena Pasqualetto, Brenda L. Volling, Laura Dal Corso

**Affiliations:** ^1^ Department of Philosophy, Sociology, Education and Applied Psychology, University of Padova, Padova, Italy; ^2^ Department of Clinical and Experimental Medicine, University of Messina, Messina, Italy; ^3^ Department of Private Law and Critique of Law, University of Padova, Padova, Italy; ^4^ Department of Psychology, University of Michigan, Ann Arbor, MI, United States

**Keywords:** return to work after maternity, Rigid Maternal Beliefs Scale, online psychological support, recovery experiences, anxiety, stress, motherhood, well-being

## Abstract

Working mothers must often balance work and family responsibilities which can be affected by rigid and irrational beliefs about motherhood. The present study had two aims: (a) to provide psychometric evidence for a shortened Italian version of the Rigid Maternal Beliefs Scale (RMBS) and (b) to facilitate mothers’ return to work after maternity leave by reducing perceptions of anxiety and stress related to rigid maternal beliefs (i.e., perceptions and societal expectations of mothers, maternal confidence, maternal dichotomy) and by teaching specific recovery strategies (e.g., relaxation, mastery experiences) to manage anxiety and stress through an online psychological intervention. Results replicated the three-factor structure of the original RMBS and showed good psychometric properties. The online psychological intervention resulted in decrease in the rigidity of maternal beliefs, perceived anxiety and stress, and increase in recovery strategies. These initial results are promising and encourage further investigation into online psychological interventions for improving the well-being of working mothers.

## Introduction

In the collective imagination, motherhood symbolizes the quintessential joyous occasion (1): the married couple transitions into a parental duo, and the child embodies the union of the two partners. The transition to motherhood is a significant developmental milestone in the lives of a great number of women. Many changes often co-occur with this transition with new challenges and responsibilities (2, 3). However, for some women, motherhood does not necessarily constitute a joyous and natural event as proposed by shared culture and media. It is a complex moment influenced by the social and relational context in which it occurs. For working mothers (WMs), the challenges and responsibilities of motherhood must be integrated into their role as workers. One of the first challenges pregnant working women may face is communicating their pregnancy status to colleagues and their supervisor. In Italy, communicating pregnancy status may be perceived negatively and be viewed as stressful and a source of concern for some women (4). Further difficulties can emerge upon returning to work (RTW) after maternity leave: the transition from being a full-time mother to a WM is considered a stressful event, characterized by compromises and changes (5). RTW after maternity leave implies a further transition for numerous women because women must integrate the RTW role as a worker into their new identity as mother. From an organizational point of view, WMs are often perceived as less committed and less reliable (6). These negative perceptions by other can lead some WMs to strive to be both good mothers and good employees, and in turn, can influence the RTW transition after motherhood, with some women finding it difficult to balance the two roles (7). Furthermore, during this delicate transition, the transition to motherhood may entail practical costs, including economic obligations and caregiving responsibilities. In Italy, some WMs find themselves having to choose between work and caring for their children, often favoring the latter and leaving the workforce. The reasons for this choice vary, such as the scarcity of public services for early childcare and the constrained labor policies that can disproportionately disadvantage young women (e.g., horizontal and vertical segregation^1^, overuse of part-time employment). In 2022, the employment rate for Italian mothers between the ages of 25 and 54 was 56.1%, whereas that of women without children was 67% (8). As such, the cultural landscape in Italy still reinforces gender differences whereby women are expected to be responsible for childcare, and in the end, this practice can perpetuate gender inequality in work-family balance. WMs may, therefore, encounter difficulties in reconciling work and family life, which may lead to widespread dissatisfaction with their lives (9). In this scenario, it is necessary to take into account the maternal leave policies and regulation for RTW in Italy, which provides the context for understanding the Italian mothers included in this study.

## Maternal leave policies in Italy and return to work

The Italian legal system recognizes female workers (especially female workers employed in paid work) and parental leave policies offer a wide range of protections relating to RTW after maternity leave. There are several legal mandates comprised both mothers and fathers in the legislative decree of 26 March 2001, n. 151. First, WMs have the right to maternity leave which must be taken from two months before to three months after the birth of the child or, if the mother's health conditions, ascertained by a medical examination, allow it, for five months entirety after the birth (compulsory maternal leave). Subsequently, if she so wishes, the woman can take additional parental leave (shared with the father) for up to six months for a total of eleven months if the father also takes leave within the first twelve years of the child's life. These leave periods from work are partly covered by an allowance paid by the National Institute for Social Security (INPS).

In addition to these basic safeguards and others prohibiting the assignment of women to tiring, dangerous, and unhealthy jobs in the months after RTW from maternity leave, the “right of return” is guaranteed. In other words, the WM has the right to keep the same job occupied before the leave and must be assigned to the same tasks she performed before giving birth or to equivalent tasks. Furthermore, female workers are not allowed to do night work due to health risks, not only during pregnancy, but also until the child is one year old. Within the child's first three years, mothers are not required to work at night, which means women must give their consent to work between midnight and 6 a.m.

Another set of guidelines seeks to facilitate the RTW gradually. Parental leave (the additional post-birth leave) can be used both daily and by the hour and women can choose to transform parental leave into part-time work. Other concessions include remote working (i.e., work that can be partially performed outside the company premises, without time constraints) and the law of 22 May 2017, no. 81, states that remote working should be prioritized if requested by women within the three years following the conclusion of compulsory maternity leave. Important safeguards are also in place regarding the matter of dismissals and resignations after the return, but also throughout the pregnancy. Dismissal is strictly forbidden up to one year after the birth of the child and the use of parental leave or leave due to the child's illness cannot be used as a reason for dismissal. As far as resignations are concerned, the legislation requires that particular procedures must be followed and validated by the Inspection Service of the Ministry of Labor to combat improper behavior by the employer. In spite of comprehensive regulation of parental leave, women's rights are not always respected, as evidenced by the jurisprudence on the matter.

## Objectives of the current research

Because the primary objective of our research program is to support WMs as they RTW, there were two aims to the current research. The first aim of this study was to advance the measurement of maternal beliefs about motherhood for a cohort of Italian women by translating the Rigid Maternal Beliefs Scale (10) and presenting psychometric evidence for a shortened Italian version of it. The shortened version of a scale is preferable whenever possible as it decreases the time needed for administration. The second aim was to help WMs to express and share their personal RTW experiences after maternity leave by exploring the meaning they attribute to this experience, through the use of a metaphor. If mothers had prior children, they reported on the most recent RTW experience. Furthermore, the current study aimed to facilitate mothers’ RTW after maternity leave by reducing maternal anxiety and stress related to rigid maternal beliefs, using an online psychological intervention that taught women specific recovery strategies and techniques to manage anxiety and stress. The research project was approved by the Ethics Committee for Psychological Research at the University of Padua, Italy (protocol n. 4695).

## Study 1

### The role of rigid maternal beliefs

There are many rigid societal expectations for women and the maternal role, such as the need to be perfect and flawless mothers in every situation, being ready to face any circumstance without doubts or hesitations, to love one's child unconditionally without ambivalent feelings at all times, and/or the expectation to be a both a good mother and housewife. All of these beliefs may have been socialized since childhood. Consequently, when a woman becomes a mother, she already carries a representation of what the maternal role entails, some of which may be based on these rigid stereotypes of motherhood, and others stemming from identification with their own mothers (11). For these reasons, mothers tend to have greater family responsibilities for childcare and household activities than their male partners (12). These rigid beliefs are constructed from shared cultural norms that may continue to shape the beliefs and experiences of WM in contemporary Italy. In so doing, these models of traditional motherhood no longer reflect the cultural changes that have occurred over the decades in the increase in women's labor force participation, and may have negative consequences for the WM, who may be viewed by some as neglecting their primary responsibility to care for their children and family. A belief is rigid and irrational when it is illogical and does not correspond to empirical reality (13). It is the cognitive expression of the unwillingness to accept an unwanted reality that hinders one's efforts to obtain something positive or prevent something negative. This is commonly expressed in the form of an absolute demands on oneself, on others, or on the world. There are four main types of irrational beliefs: demandingness (i.e., unrealistic and absolute expectations about a desired event or how people must be), awfulizing (i.e., an extreme exaggeration of the negative consequences of such a situation to make the negative event appear terrible), low frustration tolerance (i.e., derives from a demand for well-being and satisfaction and reflects the inability to tolerate suffering and discomfort), and self/other depreciation (i.e., based on the assumption that human beings can be judged and measured on a scale and that some people are worth nothing or less than others) (14). These beliefs can be reflected in every area of everyone's life, such as work (15, 16) and parenting.

Thomason and colleagues (10) have outlined several rigid beliefs about motherhood that can affect women's mental health including beliefs about societal expectations of being a mother, beliefs about anticipated maternal self-efficacy, and beliefs about child vulnerability. With respect to the first, some women's beliefs focus on their perceptions of society's expectations for what distinguishes “good” mothers from “bad” mothers. A “good” mother is always happy and positive, she must be competent and look after her children, she must never request help nor express fatigue and/or acknowledge difficulty. A “bad” mother does not accept her parental role, asks for help from her partner/parents/babysitter, and shows feelings of dissatisfaction in her role as a mother. These beliefs can transcend individual cultures and ethnicities and can be represented in cultures globally. These beliefs are mainly transmitted through the media, family members, and work interactions. When women experience the gap between society's unrealistic expectations of ideal motherhood and their daily reality where they may be challenged by parenting, these discrepancies can lead to mental health difficulties. Another rigid maternal belief centers on maternal self-efficacy and concerns whether one has the right skills or is competent enough to be a good parent. These beliefs can influence the behavior of women and affect whether they will implement behaviors they think they can carry out successfully. Maternal self-efficacy beliefs can be a protective factor for mothers when they feel competent in their maternal role, but when these beliefs are rigid, especially in relation to other mothers (e.g., I will never be able to perform my parenting duties like her; she is a “good” mother and I will never be that good), mothers may experience depressive symptoms. Even the perception of one's own children's vulnerability can represent a rigid maternal belief because a great number of mothers may believe that their children are fragile and their health and welfare completely depends on their care. As a result, they may engage in excessive or atypical behaviors that can affect children's development (e.g., frequent unnecessary medical check-ups; hypervigilance, overprotection).

The rigid maternal beliefs described above can lead to a cycle of negative thoughts that can affect mothers’ lives (e.g., if my baby cries, it means I am not a “good” mother; others expect me to be a “good” mother, if I am not, they will criticize me). Based on these rigid maternal beliefs, Thomason and colleagues (10) developed and validated the Rigidity of Maternal Beliefs Scale (RMBS), a useful tool for identifying maladaptive or rigid maternal thoughts that, if targeted during an intervention, could protect mothers from unnecessary anxiety and stress. The RMBS consists of 24 items divided into the following four dimensions: (a) *perceptions of societal expectations of mothers*—society's expectations of motherhood; (b) *role identity—*expectations of either the mother or child; (c) *maternal confidence—*confidence or efficacy of being a mother; and (d) *maternal dichotomy*,—the notion of what a good or bad mother is, specifically in relation to her parenting ability and the baby's subsequent behavior. Thomason and colleagues (10) tested the internal consistency, stability, and construct and predictive validity of the RMBS in a sample of mothers from the United States. As a first step, we tested the psychometric properties of a reduced set of RMBS items for a sample of Italian mothers.

## Method

### Participants

Participants were 256 mothers living in an urban area of northern Italy who returned to work after maternity leave. All the participants were Italian women. Most participants were between 31 and 40 years old (63.2%), 25.7% were over 41, and 11.1% were between 18 and 30 years. More than half of the participants held a university degree (66.8%); 26.4% had a high school diploma; 3.4% had a middle school diploma; 3.4% “Other” (e.g., post-lauream course). Almost all of them were employed in paid work (84.5%); 10% were freelance, 2.4% were unemployed, and 3.2% were “Other” (e.g., consultant). The majority had an open-ended contract (86%), whereas 10.9% had a fixed-term contract, and 3.1% “Other” (e.g., outsourced worker). Half of the mothers reported working full-time (40 h per week; 50.4%), whereas 45.5% worked part time (between 20 and 36 h per week); 4.1% “Other” (e.g., multiperiod schedule). Participants were asked who generally takes care of their child in times of need. Parents were indicated by 70.2% of WMs; the partner by 61.7%; the babysitter by 21.5%; “Other” (e.g., other family members) by 14.1%; and friends by 6.3% of participants.

### Measures

Rigidity of Maternal Beliefs Brief Scale (RMBBS). Rigidity of maternal beliefs was assessed with a shortened and translated Italian version of the RMBS (10). The original scale is composed of 24 items and comprises four subscales: perceptions and societal expectations of mothers (nine items); role identity (seven items); maternal confidence (four items); and maternal dichotomy (four items). The original scale items were translated into Italian by the authors. An English native-speaker translator performed a back-translation, to guarantee semantic correspondence between the English and Italian versions of the scale. To have a more manageable tool, we selected the two most representative items for each dimension. The final version produced a three-factor scale with two items each: perceptions and societal expectations of mothers (i.e., “*I feel guilty if I leave my baby with someone else in order to do something for myself”*; “*I feel guilty when I put my needs before the needs of my baby”*), maternal confidence (i.e., “*Other mothers are better able to comfort their baby”*; “*Other mothers have fewer parenting difficulties than I do”*), and maternal dichotomy (i.e., “*If my baby does not sleep well, it is a sign that I am not doing a good job as a mother”*; “*If I can't calm my baby when s/he cries, then I am not a good parent”*). Participants were asked to complete the scale with reference to their most recent experience of RTW after maternity leave. The response scale ranged from 1 (completely disagree) to 7 (completely agree). The Cronbach's alpha for the scale was .71.

Irrational beliefs at work were assessed using the Italian version of the Work-related Irrational Beliefs Questionnaire (WIB-Q) (15). The questionnaire is composed of 13 items and measures four types of work-related irrational beliefs: performance demands (three items; e.g., “*I have to be the best at work”*), coworkers’ approval (four items; e.g., “*To be happy, I must be liked by my colleagues”*), failure (three items; e.g., “*If I make a mistake, the consequences are terrible”*), and control (three items; e.g., “*I cannot cope with uncertainty at work”*). The Cronbach's alpha for each dimension was .69, .82, .82, and .80, respectively. The response scale ranged from 1 (completely disagree) to 5 (completely agree).

WMs’ mental well-being was evaluated using the General Health Questionnaire (GHQ-12) (17), a tool designed to identify general (nonpsychotic) psychiatric distress. This questionnaire inquires whether respondents have encountered specific symptoms or behaviors during the past two weeks through 12 items (e.g., “*In the last two weeks, have you lost much sleep over worry?*”). Each item is rated on a four-point scale, ranging from “less than usual” to “much more than usual”. The most commonly employed scoring methods are the bi-modal (0-0-1-1) and the Likert scoring method (0-1-2-3). In our study, we used the Likert scoring method, as recommended by Banks and colleagues (18), resulting in a total score ranging from 0 to 36. Higher scores indicate more severe psychological distress. We identified a threshold of 13/14 as the optimal cut-off point for indicating psychological distress (17). The Cronbach's alpha for the scale is .88.

### Statistical analyses

The psychometric properties of the RMBBS were evaluated through a confirmatory approach. We conducted three confirmatory factor analyses (CFAs) (i.e., one-factor model, four-factor model, and three-factor model) by means of the Lisrel 8.80 software (19). To evaluate the goodness-of-fit of the CFA models, the *χ*^2^ test was used. A model shows a good fit to data if *χ*^2^ is nonsignificant. However, because the *χ*^2^ is affected by sample size, we considered additional fit indices (20): the comparative fit index (CFI) and the nonnormed fit index (NNFI), both associated with good fit if values are ≥ .97 and with acceptable fit if values are between .95 and .97; the root-mean-square error of approximation (RMSEA), whose value ≤ .05 can be considered a good fit, while values between .05 and .08 can be considered an acceptable fit; and the standardized root-mean-square residual (SRMR), whose value ≤ .05 can be considered good, while values between .05 and .10 can be considered acceptable. Afterward, we calculated the composite reliability (CR) and the average variance extracted (AVE) indices, whose values ≥ .70 and ≥ .50, respectively, are considered satisfactory (21, 22). Lastly, missing values were considered. We excluded from analysis participants who had more than 50% missing values on a specific scale, as suggested by Hawthorne and Elliott (23). The final sample comprised 253 WMs. For the remaining missing values within a scale, we employed an item-mean imputation method (i.e., missing values on an item were replaced with the mean for that item calculated by using the scores of all study respondents who completed that item) (24, 25). Overall, 21 (.23%) missing values were imputed for the sample.

## Results

Regarding the one-factor model, the fit indices showed a bad fit to the data—*χ*^2^ (20) = 175.10, *p* = .00; CFI = .74; NNFI = .63; RMSEA = .19; SRMR = .11. In contrast, fit indices of the four-factor model indicate a good fit—*χ*^2^ (14) = 21.06, *p* = .10; CFI = .99; NNFI = .98; RMSEA = .04; SRMR = .04. Table 1 shows standardized path coefficients, CR and AVE. Standardized path coefficients were all significant and greater than .50 with the exception of items 11 and 14. Furthermore, CR and AVE reached satisfying values for all factors except *role identity*. Therefore, although the four-factor model has a good fit to the data, one of the four factors (i.e., role identity) does not show adequate psychometric properties.

**Table 1:** Standardized path coefficients, CR, and AVE for the four-factor model.

Subscale	Item	Factor loading	CR	AVE
Perceptions and societal expectations of mothers	Item 2	.69	.72	.56
	Item 9	.80		
*Role identity*	*Item 11*	*.42*	*.34*	*.21*
	*Item 14*	*.49*		
Maternal confidence	Item 18	.81	.71	.55
	Item 20	.67		
Maternal dichotomy	Item 22	.63	.76	.62
	Item 24	.92		

CR, composite reliability; AVE, average variance extracted. Non-statistically significant dimensions are in italics.

**Figure 1:**
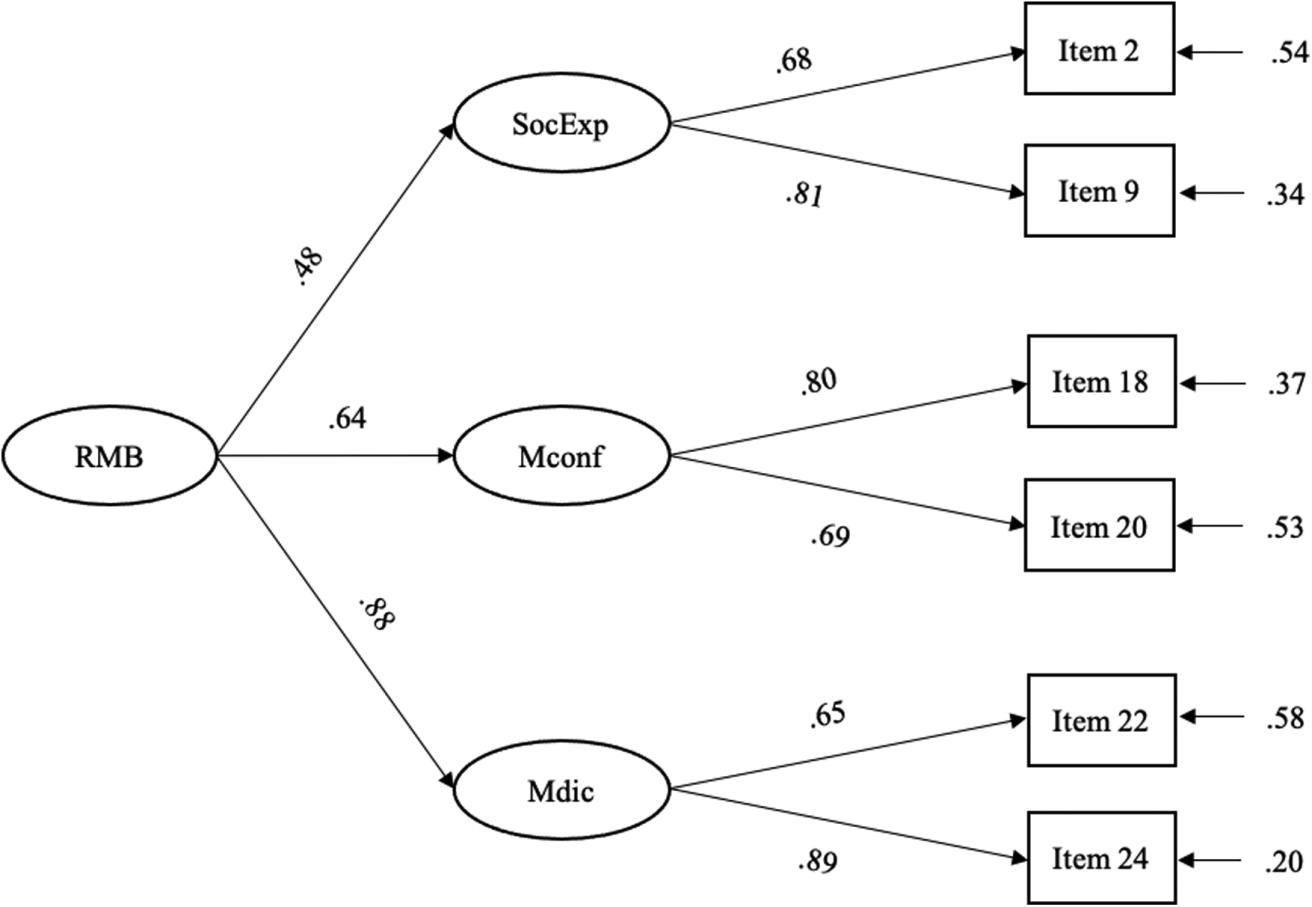
The path model. RMB, Rigidity of maternal beliefs; SocExp, Perceptions and societal expectations of mothers; Mconf, Maternal confidence; Mdic, Maternal dichotomy.

To have a measurement scale with adequate psychometric properties, we next performed a CFA of the three-factor model. The fit indices showed a good fit to data—*χ*^2^ (6) = 10.06, *p* = .12; CFI = .99; NNFI = .98; RMSEA = .05; SRMR = .03. Standardized path coefficients are all significant (Figure 1). Furthermore, CR and AVE reach satisfying values for all factors (i.e., perceptions and societal expectations of mothers—CR = .72, AVE = .56; maternal confidence—CR = .71, AVE = .55; maternal dichotomy—CR = .75, AVE = .61). Therefore, we considered the measurement model validity appropriate.

Table 2 presents the means, standard deviations, and correlations among the measured psychological dimensions. It is noted that the general level of health is good because the mean value (*M* = 9.31; *SD* = 5.85) is below the cut-off of 13/14 (17). Finally, construct validity, in terms of convergent validity, and criterion validity, in terms of concurrent validity, were examined through bivariate correlations. The positive and significant correlations between rigidity of maternal beliefs, irrational beliefs at work, and general health measures indicate appropriate validity (Table 2).

**Table 2:** Means, standard deviations, and correlations among rigidity of maternal beliefs, irrational beliefs at work, and general health measures.

	*M*	*SD*	1	2	3	4	5	6	7	8	9
1. Rigidity of maternal beliefs	2.94	.90	(.71)								
2. Perceptions and societal expectations of mothers	4.16	1.57	.71**	(.71)							
3. Maternal confidence	2.49	1.26	.67**	.11	(.71)						
4. Maternal dichotomy	2.16	1.10	.68**	.19**	.36**	(.74)					
5. Performance demands	3.22	.88	.26**	.13	.21**	.21**	(.69)				
6. Coworkers’ approval	2.46	.88	.34**	.11	.28**	.35**	.34**	(.82)			
7. Failure	2.38	1.02	.20**	.09	.16*	.19**	.41**	.40**	(.82)		
8. Control	1.71	.75	.23**	.06	.20**	.25**	.13	.29**	.25**	(.80)	
9. General health	9.31	5.85	.19**	.07	.20**	.14*	-.10	.06	.11	.10	(.88)

*M*, means; *SD*, standard deviation. Cronbach's alphas are shown in brackets.

* *p *< .05.

** *p *< .01.

## Study 2

### An online intervention for working mothers: theoretical framework

Psychoeducation can contribute to the improvement of anxiety and stress management for parents, as well as to the development of new positive resources in the person (26–29). Recovery is a dynamic process in which physical and cognitive resources are integrated to meet external demands. Specifically, it is a process in which the psychophysiological state of the person is brought back to the state (i.e., baseline) before the events that caused the fatigue. There is a distinction between internal and external recovery. Internal recovery refers to short moments of relaxation within the working day, during short breaks, whereas external recovery refers to those relaxation actions outside the organizational context (e.g., on the weekend, on vacation) (30, 31). Among the main theories used to explain the recovery process, the Conservation of Resources Theory—COR theory (32) and the Effort-Recovery Model—E-R model (33) are prominent. The first is a motivation theory that explains human behavior with respect to the need to acquire, maintain, and protect resources for survival. Acquired resources can be used to respond to stressful events or can be set aside in a “reservoir of sustaining resources” for future needs. It is the maintenance of these resources (personal, social, material) that allows people to perceive themselves as capable of facing challenging situations. As highlighted by Hobfoll and colleagues (34), the COR theory follows a series of principles and corollaries. At the basis of the theory, there is the will to obtain, maintain, increase, and protect those resources that are central to everyone's life. However, the loss of resources is disproportionately more salient than their acquisition. When people are in a state of loss of resources, obtaining new resources assumes higher importance and they are forced to invest resources for their protection. If people lose all their resources, they enter a defensive phase to protect the self. It follows that those individuals with a large number of resources will be less vulnerable to their loss than those with fewer resources. Furthermore, resources are used to cope with stressful events, so when they are depleted, stress occurs. Therefore, obtaining new resources, a process that develops slowly over time, allows people to cope with stress.

The E-R model (33) focuses mainly on the consequences of an individual's workload. High work demands and excessive workload bring on fatigue, which is understood to be a psychophysiological state characterized by low energy, high irritability, and a lack of motivation to implement new efforts. The concepts of energy and fatigue appear to be predominant in the model. According to the E-R model, there are different types of fatigue: (a) acute fatigue, usually of short duration; chronic fatigue associated with stress (i.e., exhaustion, burnout); (b) physical fatigue, associated with muscle fatigue; and (c) mental fatigue, associated with cognitive fatigue, characterized by resistance to further activity. The notion of recovery refers to the reduction or elimination of symptoms of fatigue and the restoration of energy levels. Restoring the energy level allows one to accept new job requests. However, if the energy level is not restored, this can lead to irreversible consequences related to the psychophysical health of the person. At the basis of this model, there is an emphasis on not exposing oneself for prolonged periods to demanding workloads and to recover lost energy.

Beck's cognitive model (35) provides a conceptual framework for identifying possible risk factors for maternal stress and anxiety that could be addressed during an intervention. According to this model, the relationship between life events and emotional experiences is mediated by cognitive processes. Maladaptive emotional responses, such as stress and anxiety, result from systematic biases in these cognitive processes. Thus, cognitive biases confer a vulnerability to symptoms of stress and anxiety in the context of potentially stressful life events (36). According to the cognitive model, rigid beliefs about motherhood could function as a specific cognitive risk factor for perinatal and postnatal stress and anxiety (37), which is why we focused on these rigid cognitive beliefs when designing an online intervention for mothers to alleviate stress and anxiety, and to facilitate recovery.

### Promoting recovery experiences from stress and anxiety

As noted earlier, the recovery process allows the person to restore and recover from the psychophysiological state altered by the stressful event. Sonnentag and Fritz (30) propose a series of strategies, called recovery experiences, useful for starting the recovery process, which include psychological detachment, relaxation techniques, mastery experiences, and the perception of control. Psychological detachment from work is understood as the individual's perception of totally disengaging from work, putting distance between themselves and the typical activities of the job (e.g., reading or replying to emails). However, detachment goes beyond the mere physical absence from the workplace, it is also necessary to “forget” about one's work in psychological terms. According to the E-R model, energy recovery occurs when work demands ease. Consequently, when individuals psychologically detach themselves from work not only physically, but also (and above all) mentally, chances of energies being restored increase.

Relaxation techniques are characterized by a reduced state of arousal and an improvement in positive affect. The state of relaxation can be achieved through a series of activities deliberately chosen by the individual, the goal of which is to achieve relaxation of body and mind (e.g., progressive muscle relaxation, deep breathing, meditation). What unites these activities is the minimum request for psychophysical effort, necessary for the reduction of arousal and the improvement of positive affectivity, two crucial aspects in everyone's life given that prolonged activation can induce stress and psychophysical discomfort. Relaxation experiences, therefore, help people reduce discomfort associated with stress.

The term mastery experience (i.e., those experiences relating to the mastery of a skill) refers to those extra-work activities that allow one to focus on other areas of life, providing stimulating experiences and learning opportunities (e.g., learning new languages, practicing scuba diving). Although mastering a skill is not an easy task and may require effort and sacrifice, such experiences nonetheless are a valid strategy for starting the recovery process because according to the COR theory, they allow for the acquisition of new internal resources useful for dealing with moments of stress.

Finally, the possibility of controlling one's free time and aspects of one's life can also represent a valid strategy useful for activating the recovery process. In general, control can be defined as a person's ability to choose an action between two or more possible alternatives. In this case, we refer to the possibility of choosing which activities to carry out during one's free time, as well as how and when to do them. The possibility of control is associated with positive reactions on the part of the person and can lead to a positive re-evaluation of a potentially stressful situation because the perception of discomfort is reduced and that of psychological well-being increases. The extensive body of literature examining work recovery provides evidence for the significant impact of engaging in work recovery experiences on preserving employees’ well-being, including the reduction of stress and anxiety levels (38–40).

### Online psychological interventions

The increasingly massive use of technology has led to the digitization of a great number of professions, including psychologists. Online psychological interventions have several advantages, including accessibility, because new technologies eliminate barriers, both architectural and those relating to stigma and prejudices. Another aspect of accessibility includes dematerialization of place and the canonical office hours, which allows inclusion of the requests of atypical workers (e.g., night workers, soldiers on international missions) (41). This flexibility also favours continuitation of the professional relationship, as the psychologist can maintain a connection even in those cases in which users or professionals change positions or in those cases in which social interactions are reduced (e.g., during the Covid-19 pandemic). Over the years, several studies have focused on the effectiveness of online psychological intervention: the results of some systematic reviews and meta-analyses detect no differences in effectiveness between face-to-face interventions and those delivered online (42, 43).

Online psychological interventions have also proved to be effective in supporting women in the perinatal and postnatal periods. Several studies have reported a significant reduction in anxiety, stress, and depressive symptoms compared to the control group (44–46). An example of an online psychological intervention is the MumMoodBooster (MMB) intervention (47, 48), an internet cognitive-behavioral therapy (iCBT) program aimed to support mothers in the treatment of anxiety and postpartum depression. Early and affordable interventions are often needed, and e-health has the potential to play a critical role in the transformation of health care (49).

## Method

### Participants and procedures

**Figure 2:**
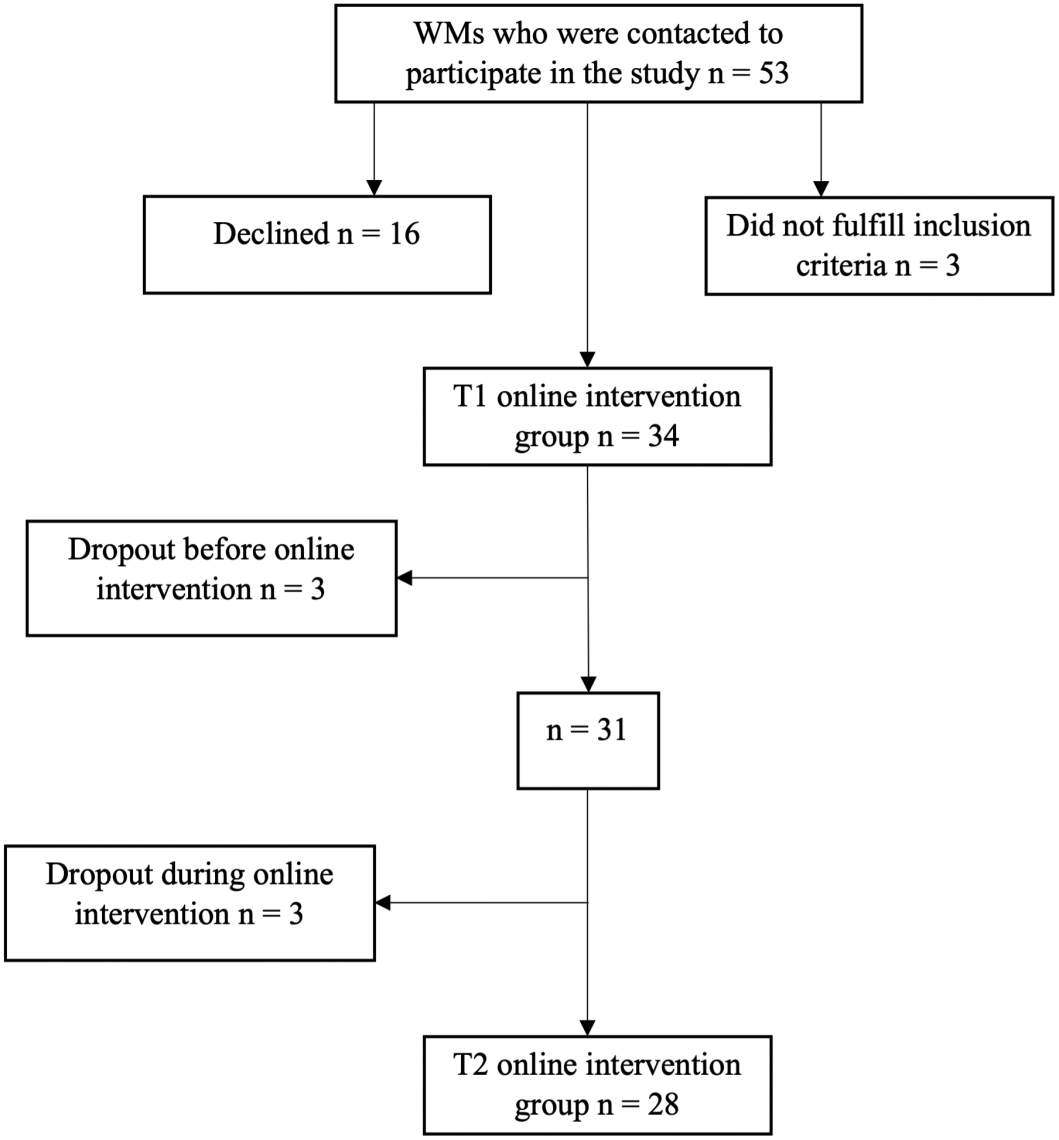
Flow chart showing WMs’ inclusion and dropout.

Statistical power analyses were used to calculate the sample size. With a medium effect size of .50 for our study, a level of significance set at *α* = .05, and a study power set at 80%, 34 participants were required to conduct the intervention study (50). Because this represented the minimum sample size needed, 53 WMs were asked via mail to participate in this second study. Participants were recruited for the online intervention if they met the following criteria: (1) had returned to work after maternity leave; (2) had at least one child (newborn—6 years old). These criteria are in line with the main aim of the study (i.e., to examine women's experiences of returning to work after maternity leave). It is well established in many countries that the burden of care shortly after birth and in general, is especially high for mothers compared to fathers. Furthermore, there are few resources and accessibility to high-quality childcare in Italy, making it challenging for parents to find infant care after the birth of an infant. Thirty-four of those 53 responded to the email and agreed to participate. All participants were Italian WMs living in an urban area of northern Italy. Most participants were between 31 and 40 years old (67.6%), 23.5% were over 41, and some were between 18 and 30 (8.8%). Almost all were employed in paid work (97.1%), while one was a freelancer (2.9%). The majority had an open-ended contract (91.2%), whereas the rest had a fixed-term contract (8.8%). Seventy-six point five percent of participants worked full-time, 23.5% part-time. Specifically, almost a third of the WMs (32.2%) worked as healthcare professionals (i.e., 25% nurse; 3.6% physiotherapist; 3.6% dietician); 21.4% worked as managers in various sectors (i.e., pharmaceutical, catering, teaching, quality, legal); 21.4% worked as office workers; 17.9% worked as teachers in middle and high schools; 7.1% worked as sales assistants. The majority of participants (92.9%) were married or cohabiting, while 7.1% were divorced or separated. Half of the WMs participating in the study had two children (50%), 35.3% had one, and 14.7% had three or more. Participants were asked who generally takes care of their child in times of need. More than a third of the participants ask their parents for help (39.3%); 32.1% ask their partner for help; 14.3% prefer to hire a babysitter; 10.8% ask for help from other family members (e.g., aunt, grandparents, in-laws); 3.6% ask for help from local services (e.g., nursery schools). All participants were duly informed that participation was anonymous and voluntary. Although 34 WMs were initially recruited, the final group consisted of 28 participants, which was due mostly to mother's own health concerns, the health of their children, or their workload (Figure 2). Because the final sample size was 28, a sensitivity power analysis was conducted to detect the effect size. With a sample size of 28, a significance level of .05, and a desired power of .80, the effect size our study could detect is .55 (50).

After providing their informed consent, participants were administered the self-report measures described below. Later, the online intervention began. The self-report questionnaires were used before (T1), and immediately after the intervention (T2). State anxiety was measured before and after each session of the intervention. Each participant was identified by an ID number to aggregate their data and preserve anonymity. All data were collected online through a Google Forms link sent by email. There were no missing data.

### Intervention

An online psychological intervention was presented to a group of WMs. Our intervention, just like other similar interventions (51, 52), was implemented in keeping with the COR theory and the E-R model (32, 33). The protocol had already been effectively applied in other organizational contexts (53, 54) through the use of virtual reality (55). The intervention consisted of four sessions, lasting approximately 50 min each, held once a week. Each session began and ended with a short interview transcribed by the researchers. The first session, consistently with the recovery experiences (i.e., relaxation techniques) of the E-R model, focused on *diaphragmatic breathing*, a type of deep breathing involving the diaphragm that is straightforward and can be used anytime, anywhere (56).

The second session focused on past successes and achievements, which activates more positive emotional responses, because of the possibility of perceiving future success. After a brief review of the diaphragmatic breathing technique, the exercise *Three Good Things* began, which consisted of asking the participant to describe two past successes, one in the personal sphere and the other in the workplace. Participants were then asked to focus on the positive aspects associated with the success experience and the emotional responses it generated. This was followed by a request to reflect on the consequences associated with this event (57). In line with the COR theory, focusing on the positive aspects represents a useful resource for coping with moments of stress.

*Body focus* or body scan was the technique taught during the third session, which focused on the physical aspects of stress. Again, this is a technique consistent with the recovery experiences (i.e., relaxation techniques) of the E-R model. Body focus is a mindfulness-based technique and consists of paying attention to the present moment intentionally and in a non-judgmental way. Specifically, the exercise involved the gradual and deliberate shifting of awareness through different parts of the body and detecting any sensation present. Body focus allows one to regulate emotions and develop adaptive responses to anxiety, thus reducing stress (58).

Finally, the fourth session focused on future positive thinking. After a brief review of the diaphragmatic breathing technique, the exercise *Best Possible Self* began, which involves thinking about the best ways in which one's life could develop in the future. Specifically, participants were invited to reflect on the best possible ways in which their lives could develop in three main domains: personal, relational, and work. For each domain, they were required to reflect on their desires, the concrete objectives that they wished to achieve, and the skills they deemed necessary to achieve them. These skills, in line with both the COR theory and the E-R model, in terms of control, will help mothers put into practice concrete actions to build new skills to achieve goals and results (59). Furthermore, all the techniques used in the four sessions were consistent with the E-R model, in terms of mastery experiences as they involved the learning of a specific technique to be used independently in times of need. At the end of each session and WMs had left the session, we sent two worksheets by email, one summarizing the session content and the other asking for feedback that would be useful for the following session.

### Measures

Mothers completed an online self-report questionnaire that assessed the following psychological dimensions.

At the beginning of the questionnaire, all participants were invited to describe their RTW after maternity leave metaphorically by completing the sentence “My RTW was … because …”. Each metaphor was identified by the same ID number of participants.

Rigidity of maternal beliefs was assessed through our Italian shortened version of the RMBS (10), named RMBBS and described in Study 1. The Cronbach's alpha for the scale is .80.

Recovery experiences were assessed through the Italian version of the Recovery Experience Questionnaire (60). The scale is composed of 16 items and comprises four subscales with four items each: psychological detachment (e.g., “*I forget about work*”); relaxation (e.g., “*I use the time to relax*”); mastery experiences (e.g., “*I seek out intellectual challenges*”); control (e.g., “*I feel like I can decide for myself what to do*”). The response scale ranged from 1 (completely disagree) to 5 (completely agree). The Cronbach's alpha for the scale is .86.

State-Trait Anxiety Inventory (STAI-Y) (61) assessed maternal anxiety. It is composed of 40 items and comprises two subscales with 20 items each: state-anxiety (e.g., “*I feel secure*”); trait-anxiety (e.g., “*I get in a state of tension or turmoil as I think over my recent concerns and interests*”). The response scale for state-anxiety ranged from 1 (not at all) to 4 (very much so). The response scale for trait-anxiety ranged from 1 (almost never) to 4 (almost always). The Cronbach's alpha for the scale is .89.

Perceived stress was assessed through the Italian Perceived Stress Scale (I-PSS-14) (62). The scale is composed of 14 items and comprises two subscales with seven items each: negative (e.g., “*In the last month, how often have you felt unable to control the important things in your life?*”); positive (e.g., “*In the last month, how often have you felt dealt successfully with day-to-day problems and annoyances?*”). The response scale ranged from 0 (never) to 5 (very often). The Cronbach's alpha for the scale is .78.

### Data analysis

Grounded theory guided the analysis of metaphors (63). In the inductive phase (64) the transcripts were read several times to identify all the metaphors used by the participants to describe RTW. In particular, all the transcripts were read by two researchers. Key emerging themes were developed by repeatedly studying the transcripts and considering possible meanings and how these fit with the developing themes.

Regarding the psychological dimensions measured during the online intervention, following preliminary analyses examining whether variables were normally distributed (Kolmogorov-Smirnov test and Shapiro-Wilk test), we conducted parametric and non-parametric analyses to perform comparisons within groups at T1 and T2 (using *t* tests for paired samples and Wilcoxon tests, respectively). Data were analyzed using IBM SPSS software version 27 (65).

## Results

### Qualitative analysis

Before starting the online psychological intervention, WMs were asked to describe their RTW experience after maternity leave metaphorically. We chose metaphors because metaphorical language can reach people in ways that the literal language does not, bringing otherwise dormant thoughts and emotions to light (66). Four key themes associated with RTW emerged: enhancement, fatigue, challenge and adventure, and dramatic events. The literature discussed in this section pertains to the meanings rather than the metaphor concept.

Ten women described RTW after maternity leave as enhancement, because it allowed them to feel like women and not just mothers. WMs seemed to feel a strong desire to combine the two roles. This perception is in line with prior literature (67, 68) which often notes how RTW, if managed appropriately, can represent a positive experience that enhances women's identity. Perceiving themselves as able to integrate the two roles helps WMs to recognize a set of skills that allows them to navigate between the two identities in the desired way. For some women, the change was perceived as “amazing” because it allowed them to express themselves in the best possible way and not just in one role, as either mother or worker. The personal and organizational contexts played an essential role in promoting a harmonious integration between the two roles (i.e., mother and worker) (69, 70).

*My RTW was like when the heat returns in spring and we can wear light weight clothes again, or when the cold arrives and we switch to wool sweaters because I was able to put into play and test my skills that I had set aside, but which instead are part of me and contribute to making me feel fulfilled.* (ID: 14)

*My RTW was a breath of oxygen because, despite the joy of becoming a mother, a woman also needs the gratification of work*. (ID: 21)

There was also a general perception of RTW as tiring, because, in addition to the obstacles that may characterize this delicate phase, WMs had to face the restrictions associated with the Covid-19 pandemic, in which a large number of them had to work from home. In this situation, the boundaries between work and family became permeable, generating misunderstandings with supervisors, colleagues, and the partner. The perception of fatigue was reported by eight mothers in the period following childbirth. Although this perception may decrease over time, for many mothers, this was not the case, especially when they RTW after maternity leave. Fatigue can limit women's engagement in daily activities, in their roles as both workers and mothers (71, 72), as the quotes below demonstrate. Organizations can support WMs through conciliation strategies (e.g., flexible hours) that allow them to start the recovery process (33).

*My RTW was a slightly uphill walk […] because I worked long hours.* (ID: 6)

*My RTW was an obstacle course, often unexpected and unclear, also due to the difficult management of continuous quarantines and misunderstandings both with the supervisor and with my husband.* (ID: 7)

However, despite the difficulties, RTW was also perceived by five mothers as an adventure or a challenge that contributed to women's personal growth. There were various challenges that WMs were called upon to face. First, they often searched for a good balance between work and family demands. In this context, the ability to adapt to the new context took on importance by recognizing one's own strengths and limits at work, trying to curb the tendency to test oneself to prove to others that one was a competent and trustworthy worker. No less important were the daily, personal, and organizational challenges (e.g., physical difficulties, barriers to breastfeeding), which may require for some women the acquisition of new resources. Here are some of the responses from two mothers (67, 73).

*My RTW was stepping on a scale because it was a constant oscillation trying to find a balance between home, work, and my needs.* (ID: 18)

*My RTW was a great new adventure because I learned to manage time and emotions […].* (ID: 20)

Finally, RTW was on occasion perceived as an experience characterized by helplessness, loneliness, and dramatic events for five WMs, as well as the perception of feeling at the mercy of events, without being in control of the situation. The transition to motherhood can generate uncertainty and feelings of inadequacy, making the reorganization of family life and the management of the new work situation even more complex (74), as this mother voiced.

*My RTW was a sad confirmation because I fell back into the same toxic dynamics present before the pregnancy, with the aggravating circumstance of having the thought of the long daily separation from the child*. (ID: 28)

At the end of the online psychological intervention, during the second administration (T2) of the self-report questionnaire, the participants were asked to describe how they felt at work following the intervention. Five participants did not report any metaphorical content. The metaphors reported by the remaining 23 WMs were analyzed. Again, thematic analysies revealed four key themes: sense of community, engagement and passion, hostility and lack of recognition, and tirelessness.

Seven participants reported a strong sense of community at work once RTW. Specifically, mothers reported a perception of themselves as “*part of a whole*” often indispensable for the success of the work, and other times, as workers “*always present*” ready to help colleagues in times of need, promoting a positive climate with “*sweetness and color*”. The perception of a sense of community at work and feeling the support of colleagues and supervisors can represent an important resource after maternity leave, which can help WMs face this delicate transition with greater positivity (4, 75). Here are a representation of these perceptions.

*When I RTW, I feel like a cog in a machine because I see the work everyone is doing around me and I see how we are all connected*. (ID: 9)

*When I RTW, I feel like a flower in a meadow of flowers because I am part of a whole and I grow and transform within it.* (ID: 23)

Furthermore, from the metaphors reported by seven WMs, there was a strong passion for work, which was carried out “*with determination and heart*”. Moreover, there was a strong engagement in the various activities undertaken by trying to “*adapt to each situation every time a new one comes up*”. This engagement may be explained by the strong meaning that WMs assigned to their work, as well as the need for continuous intellectual stimulation and creativity. These findings are in line with the findings of Grady and McCarthy (76), who found that WMs were energetic, highly successful, and highly motivated.

*When I RTW, I feel like an athlete because I am always faced with new challenges to overcome*. (ID: 11)

*When I RTW, I feel like a plant that must always be watered to grow because I am thirsty to always learn new things […].* (ID: 21)

Another key theme that emerged related to hostility and a lack of recognition at work. Specifically, six mothers reported that they did not feel valued by their supervisors. One WM reported having suffered a demotion as the coordinator at a high school due to the burden of caring for her frequently ill daughter, leading to the loss of some her favored tasks. Moreover, the fear of making mistakes and “*of not being up to the demands*” and the perception of vulnerability was also noted. These experiences after maternity leave can represent a barrier to the success and mental well-being of countless WMs. These findings are in line with Juengst and colleagues (75), who found that numerous WMs suffering discrimination for going on maternity leave, feeling pressure from colleagues and supervisors. These unfortunate experiences can lead WMs to feel uncertainty regarding their role in the organization and to questioning their capabilities (69).

*When I RTW, I feel like a spare tire because I’m always in last place, but in the times of need they always ask me*. (ID: 5)

*When I RTW, I feel like I am invisible because even though I try to respect what I’m asked to do it seems like it doesn*’*t count for anything and my figure is useless.* (ID: 25)

The last key theme that emerged concerned tirelessness. The workload seems to be perceived as demanding, and requiring a great deal of effort from the participants. In three cases, WMs appear to respond to numerous work requests with both energy and resistance, trying to complete the work assigned to them. In particular, two of them reported that they “*never sit still*” due to the many tasks they are assigned. While, on the one hand, this tireless sense of urgency can reflect energy and enthusiasm, it can also mask WMs motivation to demonstrate to others that they are “good workers” and take their work seriously. Indeed, one participant reported feeling as if she was in “*a blender that blends quickly and by immersion*”. Langan and colleagues (77) found that some WMs after maternity felt the need to challenge themselves to prove their worth, reducing the likelihood of missing important tasks. Furthermore, they tried to work even more than expected to ensure that they are seen by colleagues as “good workers”, even giving up the possibility of accessing organizational support services (e.g., programs for accommodations based on family needs).

*When I RTW, I feel like a tornado because I never stop and I’m always full of tasks to do.* (ID: 8)

*When I RTW, I feel like a cricket because I never stay still.* (ID: 26)

### Quantitative analysis

Our next analyses looked at the results from the anxiety measures completed by mothers before and after each session of the intervention. As can be seen by the significant findings in Table 3, taking into account that WMs’ trait anxiety measured at T1 is 2.26 (SD = .56), all four sessions of the online intervention led to a reduction in state anxiety.

**Table 3:** Descriptive analyses of state anxiety and pre- vs. post-session comparison.

Sessions	T1 mean (*SD*)	T2 mean (*SD*)	t or Z	*p*
1—Diaphragmatic breathing	1.93 (.55)	1.41 (.34)	−4.754	.000
2—Three Good Things	1.88 (.56)	1.42 (.32)	−4.558	.000
3—Body focus	1.87 (.52)	1.38 (.38)	−4.625	.000
4—Best Possible Self	1.85 (.49)	1.42 (.39)	−4.544	.000

Table 4 presents the means of the variables of interest at T1 and T2, along with paired *t* tests and Wilcoxon tests, for parametric and non-parametric distributions, respectively. With the exception of the negative subscale of stress, psychological detachment, and control, all other variables showed significant changes from T1 to T2 in the hypothesized directions; specifically, there were decreases in the rigidity of maternal beliefs and perceived stress, and increases in recovery experiences.

**Table 4:** Descriptive analyses and pre- vs. post-intervention comparison.

Psychological dimension	T1 mean (SD)	T2 mean (SD)	t or Z	*p*
Perceptions and societal expectations of mothers	5.05 (1.55)	4.09 (1.84)	−3.491	.000
Maternal confidence	2.98 (1.52)	2.54 (1.39)	−2.075	.038
Maternal dichotomy	3.02 (1.54)	2.48 (1.57)	−2.315	.021
Rigidity of maternal beliefs	3.68 (1.13)	3.04 (1.30)	4.923	.000
*Psychological detachment*	*2.14 (.82)*	*2.37 (.77)*	*−1.759*	*.090*
Relaxation	2.89 (1.09)	3.30 (1.25)	−2.334	.027
Mastery experiences	2.90 (1.12)	3.37 (1.30)	−2.571	.010
*Control*	*3.87 (.94)*	*4.17 (.73)*	*−1.900*	*.057*
Recovery experiences	2.95 (.72)	3.30 (.70)	−3.613	.001
Positive subscale of stress^a^	1.75 (.47)	1.33 (.66)	4.142	.000
*Negative subscale of stress*	*2.08 (.80)*	*1.89 (.68)*	*−.929*	.353
Perceived stress	1.91 (.51)	1.61 (.53)	3.954	.000

Non-statistically significant dimensions are in italics.

^a^ High values indicate a high level of stress.

## Discussion

This study stems from the desire to facilitate mothers’ RTW after maternity leave by reducing anxiety and stress due to rigid maternal beliefs about motherhood, and by teaching specific recovery strategies and techniques so mothers can learn to manage anxiety and stress. We chose the online psychological intervention format due to its flexibility and the possibility of intervening beyond physical proximity (78). This format allowed us to reach a greater number of WMs, who must often balance work and family care.

In general, the findings support the two research aims. The first aim was to provide psychometric evidence for a shortened version of the RMBS (10). Our shortened Italian version (RMBBS) of the RMBS included six items, equally divided into three subscales (perceptions and societal expectations of mothers, maternal confidence, maternal dichotomy). Results supported the three-factor structure, with good fit indices. Furthermore, the significant and positive correlations with the dimensions of irrational beliefs at work and general health provided evidence of convergent and concurrent validity.

The second study allowed us to address our second aim and to explore the metaphorical meanings that WMs attributed to their experience of RTW after maternity leave. This was described as an intense and complex experience, with a colorful emotional connotation, that may influence both motherhood and women's work choices. Furthermore, following the intervention it seems that the participants perceived themselves as able to face family and work challenges with energy, passion, and enthusiasm. Moreover, the second study evaluated an online psychological intervention to reduce rigidity in maternal beliefs, and to teach mothers useful recovery strategies for managing anxiety and stress consistent with theory and the extant literature (37, 38, 40, 79, 80). The results were promising in showing that a four session intervention reduced mothers’ reports of anxiety and rigid maternal beliefs.

## Limitations and future directions

However, there are also some limitations to this research. Although the RMBBS was used satisfactorily in the intervention, the three-factor structure was different from the original four-factor structure of Thomason and colleagues (10). This could be due to both the fact that there were fewer items used or to the cultural context and the fact that Italian maternal leave policies are dramatically different than maternal leave policies in the United States. Moreover, the perceptions and societal expectations of mothers rigid beliefs subscale, while demonstrating good psychometric properties (CR = .72; AVE = .56), requires further investigation, especially in relation to other constructs, owing to fewer significant correlations with the other dimensions pertaining to irrational work beliefs that were examined in study 1. Although we were able to show a reduction in both anxiety and rigid maternal beliefs with the online psychological intervention, the sample of WMs was relatively small and we did not include a control group or a longer-term follow-up assessment post-intervention to ascertain the effectiveness of the intervention across a year or more. Furthermore, the sample only represents a small group of WMs in Italy, so findings may not generalize outside the specific characteristics of the sample. Women in the current research were predominantly Italian women, mostly married, and working in the tertiary sector, so future research will need to expand both the use of the RMBBS and the intervention with single mothers, including those of other ethnicities, working in other working sectors. Future research will also need to evaluate the RMBBS in other cultural, social, and regulatory contexts and to consider the rigid beliefs of other caregivers, such as fathers.

Today, we see men in different traditional and non-traditional parenting roles (e.g., single fathers) and families where fathers are the stay-at-home parents taking care of their children (81). Future research needs to explore these men's experiences and perceptions when their family and parenting roles violate society's expectations of who should be the stay-at-home parent to care for children. More research is needed to understand better the importance of the paternal role for the development of men's identity throughout their lives and their different experiences with respect to their relationships with their children, partners, and society. Furthermore, in the future, the effectiveness of the intervention could be increased by proposing randomized controlled trials with a larger group of WMs, which could also include fathers, and to provide a follow-up that allows evaluation of the effectiveness of the intervention over time. Other individual characteristics of participants (e.g., perfectionism, the tendency to work excessively and compulsively) (82, 83) may need to be considered given the different societal expectations for men and women when it comes to work and family roles. Although the difficulties experienced in recent years due to the Covid-19 pandemic are not as salient, the effects of a global pandemic have changed how people work and raise their families. One of the biggest organizational changes for workplaces has been a greater adoption of remote working, which was, by necessity, the primary mode of working during the pandemic for most people in professional positions. However, not all workers were able to work from home, during the emergency period the healthcare personnel had to face the pandemic firsthand, exposing themselves to a series of risks, not only relating to a greater risk of contracting the Covid-19 virus, but also to the excessive workload, emotional toll, and the strict rules regarding personal protective equipment (84, 85). Although working from home allowed a great number to manage the personal sphere with that of work, it also proved to be a double-edged sword for others, because the boundary between family and work can become blurred and the work role can dominate home life (86). The impact of the transition to motherhood and balancing work and family responsibilities can be explored in future studies by comparing those WMs who work exclusively in person at the workplace with those WMs who work remotely.

In conclusion, online interventions may be one of the possible corporate welfare benefits aimed to promote organizational well-being and work-life balance. Corporate welfare regards services provided by a company to its employees to improve their working conditions, personal lives, and well-being by offering several options to employees to sustain their family income, by safeguarding health, parenthood and family life, and by generating several advantages for all parties involved. Information and communication technology platforms and online support interventions may be helpful to reach a greater number of employees. Furthermore, web platforms and online interventions allow the creation of a welfare system sharing experiences and best practices that can be extended to less-experienced companies. The role of new technologies is fundamental to trigger positive change and provide easy access to corporate welfare for all enterprises (87).

## Data Availability

The original contributions presented in the study are included in the article/Supplementary Material, and further inquiries can be directed to the corresponding author.

## Footnotes

^1^Horizontal segregation refers to the limited presence of women in certain working sectors and, within these sectors, the limited presence in specific areas and tasks. Vertical segregation, on the other hand, refers to the various obstacles and barriers that make women's career paths extremely difficult which work to effectively excludie them from advancement in top positions.
